# Study of the Effect of Calcium Substitution by Magnesium in the Vitreous System 3P_2_O_5_-2K_2_O-(1 − x) CaO-x MgO

**DOI:** 10.3390/ma13112637

**Published:** 2020-06-09

**Authors:** Tariq Labbilta, Mohamed Mesnaoui, Hakima Aouad, Younes Abouliatim, Mehdi Khouloud, Loubna Abielaala

**Affiliations:** 1 Laboratory of Inorganic Materials Science and Their Applications: Chemistry of Condensed Matter and Environment Team, Chemistry Department, Faculty of Sciences Semlalia, Cadi Ayyad University, Marrakech 40000, Morocco; mesnaoui@uca.ac.ma (M.M.); aouadh@uca.ac.ma (H.A.); abielaala_loubna@yahoo.fr (L.A.); 2 Laboratory of Materials, Processes, Environment, and Quality, National School of Applied Sciences of Safi Cadi Ayyad University, Safi 46000, Morocco; abouliatim.younes@gmail.com; 3 Fertilizers Unit Mohammed VI Polytechnic University–OCP Group, Jorf Lasfar 24025, Morocco; m.khouloud@ocpgroup.ma

**Keywords:** phosphate, fertilisers, glass, chemical durability, environment

## Abstract

Phosphate glasses have potentially interesting properties that can be used in various applications. Recently, different studies are focusing on their dissolution behaviours that can be modified to suit some environmental applications, such as controlled-release fertilisers. In this work, magnesium had been suggested to improve the glass durability of 3P_2_O_5_-2K_2_O-(1 − x)CaO-xMgO glasses (0 ≤ x ≤ 1). Indeed, its effect on glass structure, thermal properties and most important dissolution behaviours were studied, in order to evaluate their suitability of being used as controlled-release fertilisers. Various compositions in which calcium was partially replaced by magnesium were prepared by melting at 800 °C. The samples were characterised by differential scanning calorimetry, density measurements, X-Ray diffraction, FTIR spectroscopy and Raman spectroscopy. The dissolution behaviours were investigated using inductively coupled plasma optical emission spectrometry ICP-OES, pH measurements and SEM. Substitution of calcium by magnesium reduced the glass density, owing to the lower atomic weight of magnesium compared to calcium, and caused an increase in glass transition and crystallisation temperatures. Magnesium substitution significantly improved the chemical durability of the glasses due to more covalent Mg–O bond than the Ca–O bond. This study demonstrated that 3P_2_O_5_-2K_2_O-0.3CaO-0.7MgO (x = 0.7) had a dissolution profile adequate to the criteria of controlled-release fertilisers and could be used to nourish the plants with phosphorus, potassium, calcium and magnesium.

## 1. Introduction

Glasses are a unique and extremely flexible material that can be designed to exhibit particular properties. The most familiar and, historically, the oldest types of glass are “silicate glasses” based on the chemical compound silica. The production of other types of glass, especially those based on phosphate, is limited until today [1].

Phosphate glasses have potentially interesting properties that can be used in various applications. For example, their thermo-optical characteristics as a laser host matrix for eye protection [2,3] and thermonuclear fusion [4], their high expansion coefficient for glass to metal seals in electronics [5,6], their biocompatibility as bone substitute materials [7,8], etc.

Phosphate glasses are also known by their poor chemical durability; they offer the possibility of being completely degradable in an aqueous environment [9]; this solubility can be controlled via their composition [10], which makes them of interest for use as controlled-release fertilisers [11,12].

Controlled-release fertilisers are fertilisers containing a plant nutrient in a form, which delays its availability for plant uptake and use after application, or which extends its availability to the plant significantly longer than the usual fertilisers according to the Association of American Plant Food Control Officials (AAPFCO) [13]. The effectiveness of nutrient release control is increasing the fertility of the soil, increasing nutrient use efficiency, reducing environmental problems caused by the excessive use of rapidly available nutrient fertiliser, matching nutrient supply with plant demand and maintaining nutrient availability for the entire season. 

According to the AAPFCO, fertiliser may be described as controlled-release fertiliser if the release rate of the nutrient or nutrients meets each of the following three criteria [13]:

- No more than 15% released in 24 h,
- No more than 75% released in 28 days,
- At least about 75% released at the stated release time.

For glass fertilisers, the controlled-release rate of nutrients is mainly related to the chemical composition of the glass, which can be modified to meet the above criteria [14].

Ternary phosphate glasses, containing P_2_O_5_, CaO and K_2_O, have relatively low chemical resistance, being very soluble in aqueous media [15]. Various modifying oxides, such as Fe_2_O_3_ [16], ZnO [17] and CuO [18], have been previously suggested to improve phosphate glasses’ durability. Elements, such as zinc, copper or iron, are nutrients that are absorbed and found in lower concentrations in plant tissues (micronutrients or trace minerals). However, in higher concentrations, these elements are frequently toxic and provoke adverse effects, which will limit the rate incorporated of these elements in the vitreous matrix in order to control its dissolution.

Magnesium is an essential plant nutrient; it is one of the macronutrients, along with nitrogen, phosphorus, potassium and calcium, required for balanced crop nutrition. Often overlooked, Mg deficiencies can lead to reduced crop growth and yield. Besides, magnesium has been shown to be efficient to stabilise some phosphate glasses network; for example, D.S. Brauer [19] showed that increasing MgO amounts in glasses of the system P_2_O_5_-CaO-MgO-Na_2_O-TiO_2_ caused a significant decrease in glass solubility.

This work aimed to study the effect of calcium substitution by magnesium on physical properties, glass structure and, most importantly, the chemical durability of 3P_2_O_5_-2K_2_O-(1 − x)CaO-xMgO glasses for 0 ≤ x ≤ 1, to evaluate their suitability of being used as controlled-release fertilisers that nourish the plants with phosphorus, potassium, calcium and magnesium.

## 2. Materials and Methods 

### 2.1. Glass Synthesis

**Figure 1:**
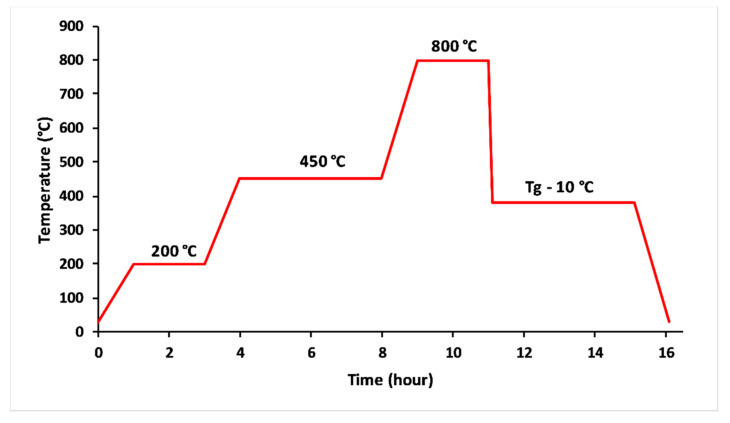
Thermal profile used to elaborate 3P_2_O_5_-2K_2_O-(1 − x)CaO-xMgO glasses.

The glasses were obtained from homogenised mixtures of CaCO_3_, K_2_CO_3_, NH_4_H_2_PO_4_ and MgO reagent grade powders. The raw materials were drily milled to a fine powder using an agate mortar. The finely ground mixture was thermally treated in alumina crucibles at 200 °C for 2 h and 450 °C for 4 h to remove NH_3_, H_2_O and CO_2_ from the decomposition of NH_4_H_2_PO_4_ and CaCO_3_ [20], as it is shown in Figure 1. The melting stage lasted 2 h at 800 ° C. The melts were quickly poured out into a carbon mould. The obtained glasses were annealed at 10 °C below their transition temperature for 4 h, then slowly quenched to room temperature. The amorphous character of the glasses was confirmed by X-ray diffraction analysis (PANAnalytical XPERT diffractometer working at 40 KV/200 mA, the angular range 10–70° (2θ) was scanned with a step size of 0.07°(2θ) and counting time of 5 s/step). The content of phosphorus, potassium, calcium and magnesium in the melted glass compositions was analyzed by inductively-coupled optical emission spectroscopy (ICP-OES Ultima Expert, Horiba).

### 2.2. Thermal Analysis

Transition temperature (Tg) and crystallisation temperature (Tc) of the melted glasses were recorded in the temperature range of 20–800 °C using a differential scanning calorimetry analyser (SETARAM DSC121 Analyser) with a heating rate of 10 °C/min. The sample consisted of a few milligrams of powder placed in a platinum crucible, while a second empty crucible served as a reference.

### 2.3. Density Measurements

Glass density measurements were made using the hydrostatic pressure concept by immersing the samples in diethyl-ortho-phthalate.

### 2.4. Characterisation of Glass Structure

Glass structure was investigated using Fourier transform infrared spectroscopy and Raman spectroscopy.

FTIR spectra were acquired using a spectrometer Bruker VERTEX 70, in the 400–4000 cm^−1^ domain, with a resolution of 4 cm^−1^, and 32 scans for each determination.

The Raman spectrum was obtained using Confotec MR520 Raman Confocal Microscope. The excitation source used was the 514 nm line of an Ar ion laser. The spectra were obtained in the range 400–4000 cm^−1^ over an average of 128 scans and 1.0 s exposure time in micro Raman compartment with 10× objective.

### 2.5. Glass Dissolution

The chemical durability of each glass sample was determined from its dissolution rate in distilled water. Glasses were ground and sieved to 1–2 mm particle size. One gram of these grains was placed in a vial containing 20 mL of distilled water with an initial pH of 6.5. 

Several samples were prepared to study the release of the glasses as a function of time. Vials were then suspended in a thermostatic bath maintained at T° = 25 ± 1 °C for 1 to 35 days.

After different immersion times, corroded glass samples were removed from leachate solutions, dried in a temperature-controlled oven at 90 °C for 10 h, and then weighted using an analytic balance sensitive (± 0.1 mg) (Shimadzu AW220).

The % of weight loss was calculated using the following formula: 

% of weight loss = $\frac{W_{i}-W_{t}}{W_{i}}\times 100$, where *w_i_* is the initial weight of the sample, and *w_t_* is the weight of the sample after *t* days.

At various dissolution time points, the pH was measured using a pH meter (Adwa-AD8000). Ionic concentration (P, K, Ca, Mg) in leachate solutions was analysed by inductively-coupled optical emission spectroscopy.

Surface changes were observed by scanning electron microscope (Tescan VEGA 3 equipped with KEVEX detector (Si–Li diode) working in X energy dispersion (X-EDS)). The studied samples were metallised with carbon. The acceleration voltage was 10 kV, the acquisition time was 120 s, and the angle of the output was 30°. Glass samples intended for surface analysis were annealed, cut into parallelepiped shapes (1 × 1 × 0.5 cm^3^), with carefully polished surface, then placed in a vial containing distilled water with an initial pH of 6.5 for 3 days.

## 3. Results and Discussion

### 3.1. Glass Formation

During the preparation, the molten liquids were fluid, and the quenching was carried out without problems. Few bubbles were observed in the glasses, and all the obtained glasses were transparent and colourless.

The nominal and analysed compositions of the glasses are presented in Table 1. Differences between nominal and analysed compositions were comparable for all samples and were attributed to melting volatilisation and measurement errors.

**Figure 2:**
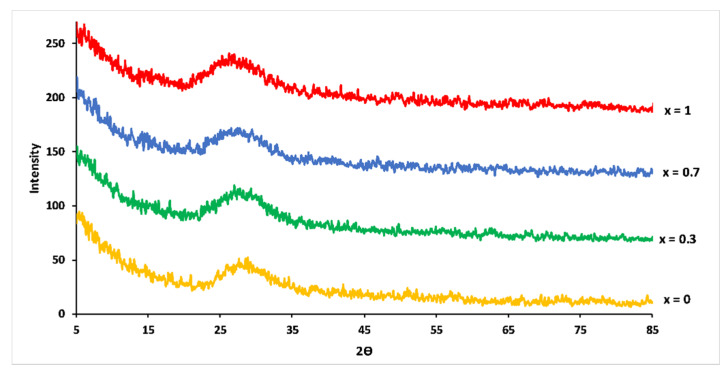
XRD patterns for 3P_2_O_5_-2K_2_O-(1 − x)CaO-xMgO glasses.

XRD pattern of the studied glasses showed that all samples were vitreous materials without crystalline phase (Figure 2). With increasing Mg substitution, maxima of the amorphous halos in XRD patterns shifted to smaller 2θ angles. This shift suggested an increase in the compactness of the phosphate network caused by the smaller ionic radius of Mg^2+^ (0.072 nm) compared to Ca^2+^ (0.099 nm) [21].

### 3.2. Thermal Behaviour

**Figure 3:**
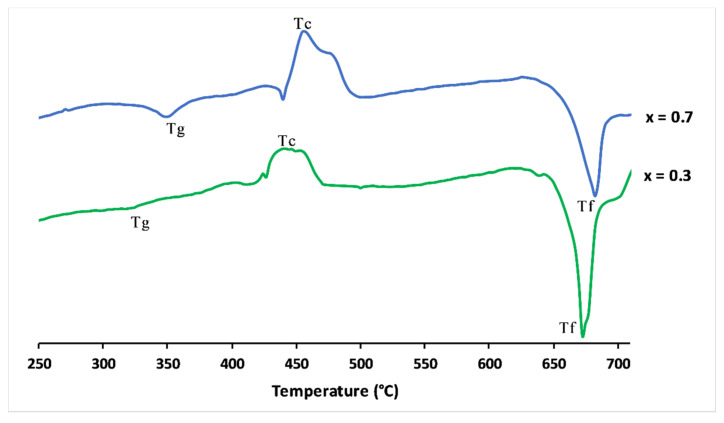
Differential scanning calorimetry curves of 3P_2_O_5_-2K_2_O-0.7CaO-0.3MgO and 3P_2_O_5_-2K_2_O-0.3CaO-0.7MgO glasses.

Figure 3 shows the differential scanning calorimetry curves of x = 0.3 and x = 0.7 glasses. The clear features were attributed to the glass transition temperature (Tg). The upward peaks were exothermic and represented the glass crystallisation temperature (Tc). The endothermic peaks defined the melting point (Tf).

**Figure 4:**
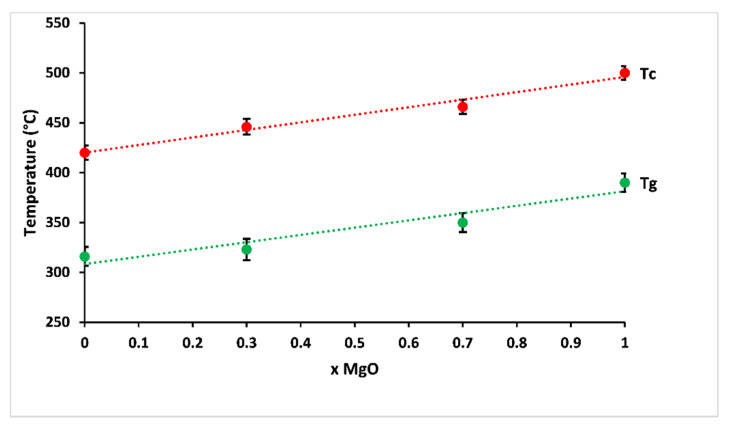
Glass transition (Tg) and crystallisation (Tc) temperatures of prepared glasses according to MgO contents.

The variation of glass transition and crystallisation temperatures, according to MgO contents, is shown in Figure 4. The studied glasses showed glass transition temperatures between 316 and 390 °C, and the crystallisation temperature increased from 420 to 500 °C with increasing magnesium substitution. This behaviour corresponded to some changes in the nature of bonding in the structural network: magnesium has a smaller ionic radius than calcium, causing a larger charge to size ratio (z/r2, where z is the valence cation, and r is the ionic radius), which makes the glass more rigid owing to more efficient cross-linking, leading to a more compact network [22]. Besides, changes in polarity of the bonds as a result of the higher electronegativity of magnesium compared to calcium are likely to affect glass transition and crystallisation temperatures of phosphate glasses [23,24,25].

### 3.3. Glass Density

**Figure 5:**
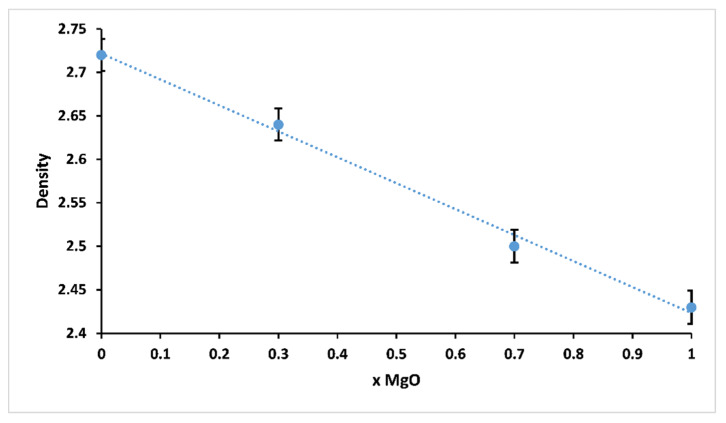
Glass density of 3P_2_O_5_-2K_2_O-(1 − x)CaO-xMgO glasses for 0 ≤ x ≤ 1.

The variation of density of 3P_2_O_5_-2K_2_O-(1 − x)CaO-xMgO glasses for 0 ≤ x ≤ 1 is presented in Figure 5.

Density is one of the effective tools to explore the degree of structural compactness of the vitreous network. It is sensitive to spatial arrangement and the nature of atoms [26,27]. Therefore, it depends on the composition of the glass. It could be clearly seen that the density of the studied glasses decreased from 2.72 (x = 0) to 2.43 g.cm^−3^ (x = 1) with increasing Mg substitution. The decrease in density was possibly due to the smaller atomic weight of magnesium (24 g mol^−1^) compared to calcium (40 g mol^−1^).

### 3.4. Glass Structure

**Figure 6:**
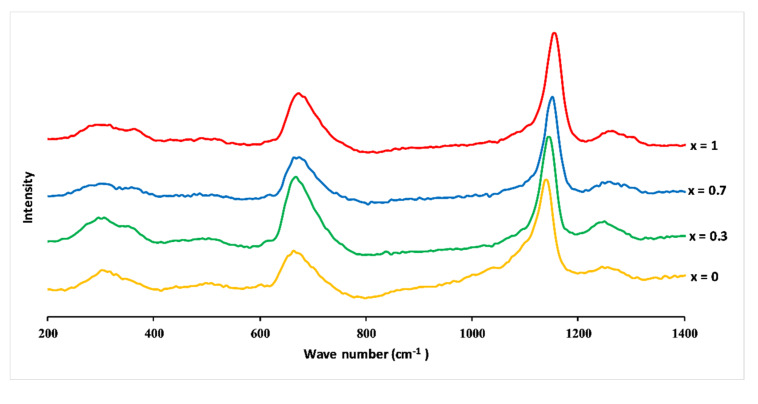
Raman spectra of 3P_2_O_5_-2K_2_O-(1 − x)CaO-xMgO glasses for 0 ≤ x ≤ 1.

Figure 6 shows Raman spectra of 3P_2_O_5_-2K_2_O-(1 − x)CaO-xMgO glasses (0 ≤ x ≤ 1). The infrared and Raman band assignments are listed in Table 2.

The spectra were dominated by peaks around 680 and 1160 cm^−1^ and revealed additional weaker bands, which could be typical, according to the literature [28,29,30], of phosphates with metaphosphate glass composition. The band at around 680 cm^−1^ corresponded to symmetric stretching vibration V_s_(P–O–P) of the Q^2^ groups (in the Q^n^ terminology, n represents the number of bridging oxygens per PO_4_ tetrahedron) [31]. The most intense peak at 1160 cm^−1^ was assigned to the symmetric stretch mode of non-bridging oxygens V_s_(PO_2_^−^), and the band at 1280 cm^–1^ was attributed to the asymmetric stretch of O–P–O, V_as_(PO_2_^−^), both in Q^2^ groups. The large feature between 250 and 400 cm^−1^ involved the bending vibrations of phosphate polyhedral, δ(P–O–P). The appearance of a low band at about 1050 cm^−1^ was due to the symmetric stretching vibration of terminal PO_3_^2−^ units, V_s_(PO_3_^2−^), and signified the formation of Q^1^ units [21,28,29,30,31,32].

Raman spectra identified Q^2^ groups as the main structural unit, accompanied by the absence of Q^3^ and a minor amount of Q^1^ groups, as expected for the metaphosphate compositions [21].

The substitution of CaO by MgO caused a shift towards the high frequencies of the most intense Raman peaks, for example, V_s_(PO_2_^−^) shifted from 1143 to 1157 cm^−1^, and V_s_(P–O–P) shifted from 668 to 679 cm^−1^.

Such changes in vibration frequency could be attributed to the covalent variation of the bond (P–O). As mentioned before, the size of the Mg^2+^ ion is smaller than the size of the Ca^2+^ ion, which means a larger field strength of Mg^2+^ ions compared to Ca^2+^. This results in a more covalent Mg–O bond than the Ca–O bond, leading to a more ionic P–O(Mg) bond than P–O(Ca) bond [31].

**Figure 7:**
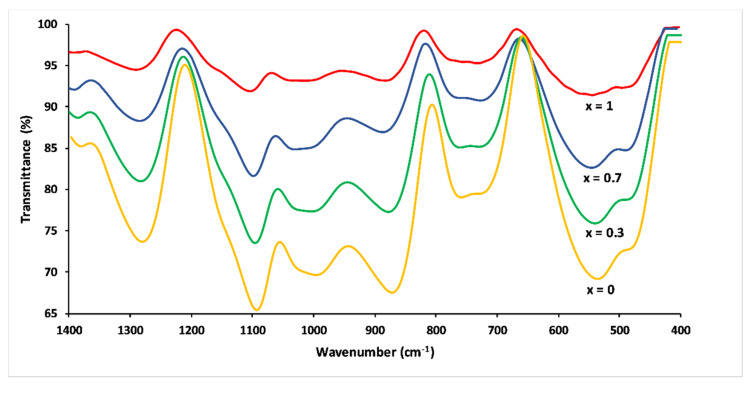
FTIR spectra of 3P_2_O_5_-2K_2_O-(1 − x)CaO-xMgO glasses for 0 ≤ x ≤ 1.

FTIR spectra of the 3P_2_O_5_-2K_2_O-(1 − x)CaO-xMgO glasses are presented in Figure 7. The IR spectra were more complex compared to the Raman spectra due to the strong band overlap. 

The band at 1290–1260 cm^−1^ corresponded to asymmetric stretching mode V_as_(PO_2_^−^) in Q^2^ phosphate tetrahedron. The FTIR band at 1148 cm^−1^ corresponded to the symmetric stretch of (O–P–O) in Q^2^ groups. The absorption bands around 1100 and 1000 cm^−1^ corresponded to the asymmetric and symmetric stretching modes—V_as_ (PO_2_^−^) and V_s_ (PO_3_^2−^)—in Q^1^ groups, respectively. The two peaks between 900 and 700 cm^−1^ were assigned, respectively, to asymmetric and symmetric stretching of the bridging oxygen atoms bonded to a phosphorus atom in a Q^2^ phosphate tetrahedron. The bending vibrations of phosphate polyhedral δ(PO_2_^−^) and δ(PO_3_^2−^) resulted in the range between 400 and 600 cm^−1^ [22,33,34].

In analogy to their Raman counterparts, the substitution of CaO by MgO caused a shift towards the high frequencies of the FTIR bands. Besides, peak intensities became much weaker with increasing Mg substitution, an effect which has also been observed by other researchers [21]. This could be explained by differences in electronegativity for calcium and magnesium. Magnesium has a higher electronegativity, resulting in a decrease in the absorption bands [21,31].

### 3.5. Dissolution Behaviour

**Figure 8:**
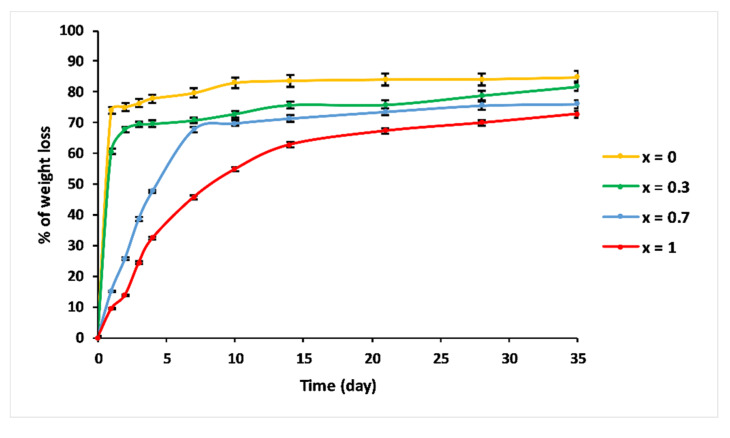
Trend of weight loss of 3P_2_O_5_-2K_2_O-(1 − x)CaO-xMgO (0 ≤ x ≤ 1) glasses versus immersion time at T = 25 °C.

Figure 8 shows the percentage of weight loss of the 3P_2_O_5_-2K_2_O-(1 − x)CaO-xMgO system glasses after being immersed in 25 °C water for 35 days. 

The initial dissolution rates V_0_, calculated from the slope of the linear approximation (V_0_ = $\frac{dm}{dt}$) of the dissolution curves, are presented in Table 3.

Compositions—x = 0 and x = 0.3—exhibited similar dissolution behaviour as a function of immersion time. The dissolution curves could be divided into two steps: Step 1, consisting of a linear range, where the weight loss increased dramatically, showing a glass network dissolution, which was not influenced by solution saturation, with initial dissolution rates V_0_ = 0.74 g/day and 0.607 g/day for x = 0 and x = 0.3, respectively. The second step is where the dissolution slowed down. This could be explained by the saturation of the aqueous solution [35].

Glasses with high Mg content—x = 0.7 and x = 1—revealed a different dissolution behaviour. As a function of immersion time, three ranges could be distinguished. For t < 7 days, the % of weight loss was almost linear, with V_0_ = 0.1 g/day and 0.064 g/day for x = 0.7 and x = 1, respectively. The 7 < t < 21 days range was characterised by a moderate dissolution. After 21 days, the dissolution curve indicated a slowing down.

The dissolution rates of these glasses depended on the MgO contents. As the MgO substitution increased, the chemical durability increased. This behaviour was due to the structural alteration of phosphate glasses, implying the creation of a stronger network that exhibited high chemical resistance in aqueous media.

The use of MgO as a network modifier provided ionic cross-linking between non-bridging oxygen atoms of two phosphate chains and increased the bond strength and chemical durability of the glasses. As mentioned previously, the improved glass resistant might be attributed to the replacement of the Ca–O–P bonds by more hydration resistance P–O–Mg bonds. As the MgO amount in the glass increased, the number of P–O–Mg bonds also increased. This explanation was in line with the results of the structural and thermal studies, which clearly indicated an increase of the phosphate network rigidity.

**Figure 9:**
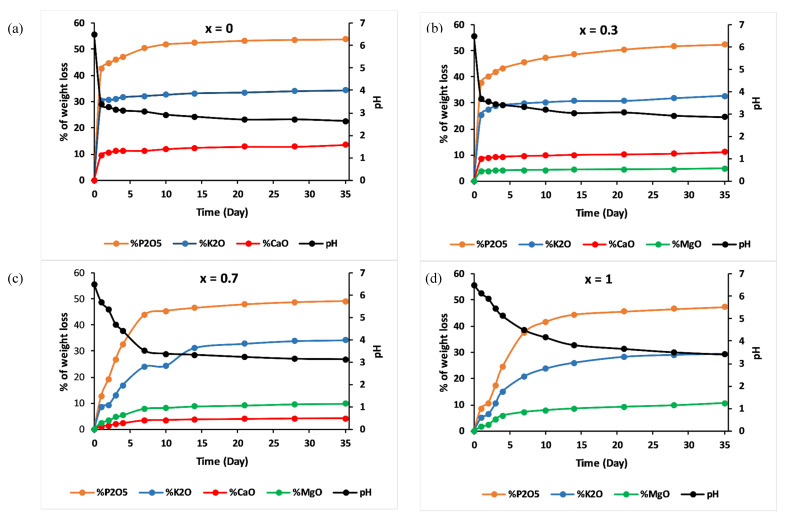
% of glass constituents analysed in the leachate solutions (cations in form of oxides) normalized to the initial glass weight and pH measurements versus time for (**a**) x = 0, (**b**) x = 0.3, (**c**) x = 0.7 and (**d**) x = 1.

Amounts of cations, in the form of oxides normalised to the initial glass weight, released from the examined glasses as determined by the ICP-OES method, and the pH measurements are shown in Figure 9.

Elemental concentrations in leachate solution increased over time, while concentrations of calcium decreased and concentrations of magnesium increased with increasing magnesium substitution in the glass.

For all the studied glasses, the presence of entire cations in the leachate solution, with a ratio equivalent to the glasses composition, led to the conclusion that dissolution was congruent, which meant that it was achieved by hydration reactions, with a two-stage mechanism: surface hydration resulting from water diffusion, then release of metaphosphate chains and the other constituents of the glass in the leachate solution [36]. The addition of MgO slowed down this mechanism: Figure 9 shows that the amount of P released into water decreased by nearly five orders of magnitude, on the first day, with an increase in the MgO content from x = 0 to x = 1.

The pH of the leachate solutions decreased from 6.5 to reach the acidic range for all glasses, then remained almost constant for periods of immersion. The decrease in pH could be assigned to the disintegration of phosphate entities toward the solution and the possibility of the formation of phosphoric acid H_3_PO_4_ [10]. With increasing MgO amounts, less phosphorus would be released in water, meaning a less significant decrease in pH. 

As dissolution tests showed low pH values, one might expect that the application of these glasses as fertilisers could acidify the soil. However, one advantage of controlled-release fertilisers is that they match nutrient supply with plant demand, which means that the amount of phosphorus released from phosphate glasses (most responsible for pH decrease) will be assimilated by plant roots. 

To evaluate the suitability of the studied glasses to the standards of the AAPFCO for controlled-release fertilisers, the % of weight loss of cations, in the form of oxides, calculated from their initial weights in the glass ($\frac{W_{t}\left(\mathrm{oxide}\right)}{W_{i}\left(\mathrm{oxide}\right)}$ × 100), after an immersion time of 24 h and 28 days, are presented in Table 4.

It could be seen that by increasing MgO contents, the % of weight loss of all glass constituents decreased:

- For x = 0 and x = 0.3 glasses, the % of weight loss was very high compared to the AAPFCO standards (up to 60% after only one day of immersion), meaning that these glasses could not be considered as controlled-release fertilisers.
- 3P_2_O_5_-2K_2_O-0.3CaO-0.7MgO glass satisfied the requirements of controlled-release fertilisers by showing results relatively similar to the criteria (P_2_O_5_, for example, had a % of weight loss of 15.7% after one day, 75.3% after 4 weeks and 76.1 after 35 days).
- For x = 1 glass, all the oxides were released in amounts below the AAPFCO standards for 24 h and 28 days, but it did not reach the threshold required by the third criteria (≥ 75% after 35 days) for most of its constituents. Moreover, this composition did not contain calcium, which is an essential element for plant nutrition.

**Figure 10:**
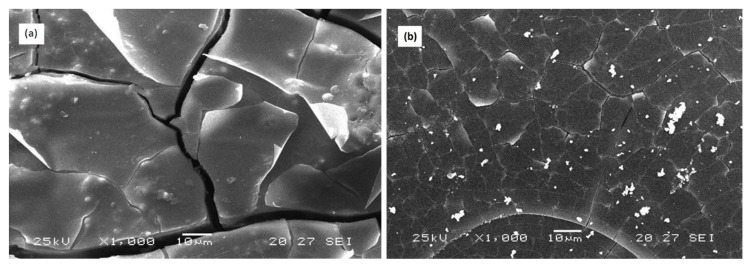
SEM micrographs of (**a**) 3P_2_O_5_-2K_2_O-0.7CaO-0.3MgO and (**b**) 3P_2_O_5_-2K_2_O-0.3CaO-0.7MgO glasses’ surface after 3 days of immersion time at 25 °C.

SEM observation of samples immersed for 72 h in the distilled water at 25 °C of the two compositions 3P_2_O_5_-2K_2_O-0.7CaO-0.3MgO and 3P_2_O_5_-2K_2_O-0.3CaO-0.7MgO are shown in Figure 10.

The micrograph (a) depicts a rough and cracked surface for the sample with x = 0.3, while the surface of the glass with x = 0.7 after corrosion (b) underwent only minor changes.

These results were in agreement with the results of weight loss of the glasses, which showed that the glass x = 0.3 had a more significant weight loss than the glass x = 0.7.

## 4. Conclusions

The effect of magnesium for calcium substitution was investigated in the system 3P_2_O_5_-2K_2_O-(1 − x)CaO-xMgO with 0 ≤ x ≤ 1.

Both calcium and magnesium belong to the alkaline earth metals group. However, the smaller atomic weight and the smaller ionic radius of magnesium compared with calcium have shown that magnesium has a completely different behaviour from calcium within the metaphosphate glass network.

From the research that was carried out, it was possible to conclude that increasing the MgO content in 3P_2_O_5_-2K_2_O-(1 − x)CaO-xMgO phosphate glasses improved their chemical durability. This result was consistent with the evolution of the thermal properties (Tg and Tc) of these glasses. 

FTIR and Raman spectroscopic investigations were recorded to determine the structural evolution of the studied phosphate glasses. One could notice the modification of the vitreous network, suggesting the strengthening of the metaphosphate chains when the MgO oxides were introduced.

According to weight loss, pH, ICP-MS and SEM results, the dissolution rates of the glasses were considerably diminished with the addition of MgO content. It could be concluded that the chemical durability of these phosphate glasses in distilled water rested on their chemical composition and structure of the glass network, especially by the degree of its cross-linking and the strength of the chemical bonds between the modifier cation (Mg^2+^) and the components of the network.

This study showed that the composition 3P_2_O_5_-2K_2_O-0.3CaO-0.7MgO met the criteria of the AAPFCO for controlled-release fertilisers. This glass could be used as fertiliser, which could nourish the plants with phosphorus, potassium, calcium and magnesium. Future research should confirm the validity of this hypothesis under greenhouse and field conditions. 

## Figures and Tables

**Table 1:** Nominal and analysed compositions of the studied glasses.

Glass	Nominal Composition (% mol)				Analysed Composition (% mol)			
**x**	**P_2_O_5_**	**K_2_O**	**CaO**	**MgO**	**P_2_O_5_**	**K_2_O**	**CaO**	**MgO**
**0**	50	33.33	16.66	0	51.57	32.67	15.74	0
**0.1**	50	33.33	15	1.66	52.62	31.79	14.21	1.39
**0.2**	50	33.33	13.33	3.33	51.06	32.88	12.96	3.11
**0.3**	50	33.33	11.66	5	51.37	32.53	10.97	5.12
**0.4**	50	33.33	10	6.66	50.81	32.76	9.02	7.39
**0.5**	50	33.33	8.33	8.33	51.95	32.90	7.82	7.93
**0.6**	50	33.33	6.66	10	51.68	32.87	5.97	9.47
**0.7**	50	33.33	5	11.66	51.40	32.86	4.78	11.94
**0.8**	50	33.33	3.33	13.33	51.65	32.74	2.87	12.73
**0.9**	50	33.33	1.66	15	51.82	32.75	1.20	14.22
**1**	50	33.33	0	16.66	50.36	31.46	0	18.17

**Table 2:** Frequency ranges (cm^−1^) and assignments of the Raman and infrared bands of the studied glasses.

x	V_as_ (PO_2_^−^), Q^2^		V_s_ (PO_2_^−^), Q^2^		V_s_(PO_3_^2−^), Q^1^		V_as_(PO_2_^−^), Q^1^		V_as_ (P–O–P), Q^2^		V_s_ (P–O–P), Q^2^		δ(PO_2_^−^)		δ(PO_3_^2−^)	
	FTIR	Raman	FTIR	Raman	FTIR	Raman	FTIR	Raman	FTIR	Raman	FTIR	Raman	FTIR	Raman	FTIR	Raman
**0**	1276	1252	1145	1142	1089	1048	989–1020	-	867	879	721–755	672	532	352	582	306
**0.3**	1278	1255	1151	1147	1095	-	991–1026	-	876	-	723–763	674	538	357	582	310
**0.7**	1282	1263	1156	1152	1099	-	997–1031	-	879	-	723–767	679	541	363	585	306–318
**1**	1284	1268	1159	1157	1101	-	997–1031	-	879	-	725–771	685	547	369	584	295–326

V_as_: asymmetric stretching vibration; V_s_: symmetric stretching vibration; δ: bending vibration; Q^n^: the number of bridging oxygens per PO_4_ tetrahedron.

**Table 3:** Initial dissolution rates of 3P_2_O_5_-2K_2_O-(1 − x)CaO-xMgO (0 ≤ x ≤ 1) glasses at T = 25 °C.

x MgO	V_0_ (g/day)
x = 0	0.74
x = 0.3	0.607
x = 0.7	0.1
x = 1	0.064

**Table 4:** % of weight loss of cations, in the form of oxides, after an immersion time of 24 h and 28 days.

		% of Weight Loss after 24 h	% of Weight Loss after 28 Days	% of Weight Loss after 35 Days
	AAPFCO Criteria	≤ 15%	≤ 75%	≥ 75%
**P_2_O_5_**	x = 0	71%	84.5%	84.7%
	x = 0.3	60%	81%	81.8%
	x = 0.7	15.7%	75.3%	76.1%
	x = 1	10.2%	71.6%	72.8%
**K_2_O**	x = 0	69.4%	81%	81.5%
	x = 0.3	59.7%	78.8%	80.1%
	x = 0.7	14.9%	74.8%	77.5%
	x = 1	11.3%	72.2%	74.2%
**CaO**	x = 0	72%	81.2%	84%
	x = 0.3	61.1%	78%	79.3%
	x = 0.7	15.2%	75.6%	76.1%
**MgO**	x = 0.3	46.2%	73.5%	77.8%
	x = 0.7	15.2%	73.8%	74.7%
	x = 1	8.2%	69.9%	73.7%

AAPFCO: Association of American Plant Food Control Officials.

## References

[1] Videau J.J., Le Flem G. (2009). Phosphate Glasses: From the Specificity of the Phosphorus Atom to the Formation, Structure and Chemical Durability of Phosphate Glasses.

[2] Seshadri M., Rao K.V., Rao J.L., Ratnakaram Y.C. (2009). Spectroscopic and laser properties of Sm^3+^ doped different phosphate glasses. J. Alloy. Compd..

[3] Weber M.J. (1990). Science and technology of laser glass. J. Non. Cryst. Solids.

[4] Yamanaka C., Nakai S., Yamanaka T., Izawa Y., Kato Y., Mima K., Nishihara K., Mochizuki T., Yamanaka M., Nakatsuka M. (1987). High thermonuclear neutron yield by shock multiplexing implosion with GEKKO XII green laser. Nucl. Fusion.

[5] Brow R.K., Kovaic L. (1994). New glasses for hermetic aluminium seals. Seal. Technol..

[6] Maeder T. (2013). Review of Bi_2_O_3_ based glasses for electronics and related applications. Int. Mater. Rev..

[7] Karadjian M., Essers C., Tsitlakidis S., Reible B., Moghaddam A., Boccaccini A.R., Westhauser F. (2019). Biological properties of calcium phosphate bioactive glass composite bone substitutes: Current experimental evidence. Int. J. Mol. Sci..

[8] Yousefi A.M. (2019). A review of calcium phosphate cements and acrylic bone cements as injectable materials for bone repair and implant fixation. J. Appl. Biomater. Funct. Mater..

[9] Reis S.T., Karabulut M., Day D.E. (2001). Chemical durability and structure of zinc-iron phosphate glasses. J. Non. Cryst. Solids.

[10] Abielaala L., Aouad H., Mesnaoui M., Musso J.A. (2011). Characterization and vitrification of fly ashes from incineration of waste of infectious risk care (WIRC). Sustain. Environ. Res..

[11] Waclawska I., Szumera M. (2009). Reactivity of silicate-phosphate glasses in soil environment. J. Alloy. Compd..

[12] Lee Y.S., Kang W.H. (2004). Structure and dissolution properties of phosphate glasses for glass fertiliser. Mater. Sci. Forum.

[13] Trenkel M.E. (2010). Slow- and Controlled-Release and Stabilized Fertilizers: An Option for Enhancing Nutrient Efficiency in Agriculture.

[14] Hazra G., Das T. (2014). A Review on Controlled Release Advanced Glassy Fertilizer. Glob. J. Sci. Front. Res. B Chem..

[15] Knowles J.C., Franks K., Abrahams I. (2001). Investigation of the solubility and ion release in the glass system K_2_O-Na_2_O-CaO-P_2_O_5_. Biomaterial.

[16] Abrahams I., Franks K., Hawkes G.E., Philippou G., Knowles J.C., Nunes T.G. (1997). 23 Na, 27 Al and 31 P NMR and X-ray powder direction study of Na/Ca/Al phosphate glasses and ceramics. J. Mater. Chem..

[17] Abou Neel E.A., O’Dell L.A., Smith M.E., Knowles J.C. (2008). Processing, characterisation, and biocompatibility of zinc modified metaphosphate-based glasses for biomedical applications. J. Mater. Sci. Mater. Med..

[18] Shih P.Y., Ding J.Y., Lee S.Y. (2003). ^31^P MAS-NMR and FTIR analyses on the structure of CuO-containing sodium poly- and meta-phosphate glasses. Mater. Chem. Phys..

[19] Brauer D.S. (2005). Degradable Phosphate Glasses and Composite Materials for Biomedical Applications. Dissertation. Bachelor’s Thesis.

[20] Omrani R.O., Krimi S., Videau J.J., Khattech I., El Jazouli A., Jemal M. (2014). Structural investigations and calorimetric dissolution of manganese phosphate glasses. J. Non. Cryst. Solids.

[21] Morikawa H., Lee S., Kasuga T., Brauer D.S. (2013). Effects of magnesium for calcium substitution in P_2_O_5_-CaO-TiO_2_ glasses. J. Non. Cryst. Solids.

[22] Lee S., Maeda H., Obata A., Ueda K., Narushima T., Kasuga T. (2016). Structures and dissolution behaviors of MgO–CaO–P_2_O_5_–Nb_2_O_5_ glasses. J. Non. Cryst. Solids.

[23] Griebenow K., Bragatto C.B., Kamitsos E.I., Wondraczek L. (2018). Mixed-modifier effect in alkaline earth metaphosphate glasses. J. Non. Cryst. Solids.

[24] Ray N.H. (1974). Composition-property relationships in inorganic oxide glasses. J. Non. Cryst. Solids.

[25] Hudgens J.J., Martin S.W. (1993). Glass Transition and Infrared Spectra of Low-Alkali, Anhydrous Lithium Phosphate Glasses. J. Am. Ceram. Soc..

[26] Sharaf El-Deen L.M., Al Salhi M.S., Elkholy M.M. (2008). Spectral properties of PbO–P_2_O_5_ glasses. J. Non. Cryst. Solids.

[27] Pascual L., Durán A. (1996). Nitridation of glasses in the system R_2_O-MO-P_2_O_5_. Mater. Res. Bull..

[28] Lai Y.M., Liang X.F., Yang S.Y., Wang J.X., Zhang B.T. (2012). Raman spectra study of iron phosphate glasses with sodium sulfate. J. Mol. Struct..

[29] Videau J.J., El Hadrami A., Labrugère C., Couzi M., Montagne L., Mesnaoui M., Maazaz M. (2007). Structural influence of alumina in Zn-Cd-Pb phosphate glasses. Phys. Chem. Glas. Eur. J. Glas. Sci. Technol. Part B.

[30] Velli L.L., Varsamis C.P.E., Kamitsos E.I., Möncke D., Ehrt D. (2005). Structural investigation of metaphosphate glasses. Phys. Chem. Glas..

[31] Karakassides M.A., Saranti A., Koutselas I. (2004). Preparation and structural study of binary phosphate glasses with high calcium and/or magnesium content. J. Non. Cryst. Solids.

[32] Kiani A., Hanna J.V., King S.P., Rees G.J., Smith M.E., Roohpour N., Salih V., Knowles J.C. (2012). Structural characterisation and physical properties of P_2_O_5_-CaO-Na_2_O-TiO_2_ glasses by Fourier transform infrared, Raman and solid-state magic angle spinning nuclear magnetic resonance spectroscopies. Acta Biomater..

[33] Nian S., Zhang Y., Li J., Zhou N., Zou W. (2018). Glass formation and properties of sodium zinc phosphate glasses doped with ferric oxide. Adv. Appl. Ceram..

[34] Makhlouk R., Beloued N., Aqdim S. (2018). Study of Chromium-Lead-Phosphate Glasses by XRD, IR, Density and Chemical Durability. Adv. Mater. Phys. Chem..

[35] Kouassi S.S., Andji J., Bonnet J.P., Rossignol S. (2010). Dissolution of waste glasses in high alkaline solutions. Ceram. Silikaty.

[36] Ma L.N. (2014). Dissolution Behaviour of Phosphate Glasses. Ph.D. Thesis.

